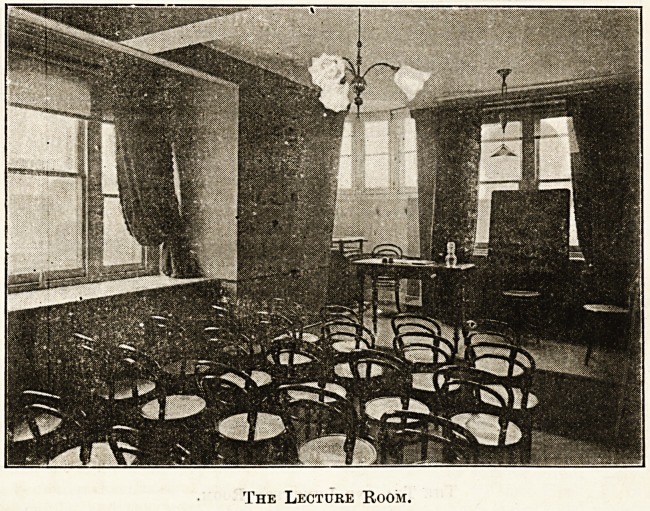# "The Hospital" Nursing Mirror

**Published:** 1897-04-03

**Authors:** 


### "The Hospital" Nursing Mirror.

Being the Nursing Section of "The Hospital."

[Contributions for this Section of "The Hospital" should be addressed to the Editor, The Hospital, 28 & 29, Southampton Street, Strand, London, W.C., and should have the word "Nursing" plainly written in left-hand top corner of the envelope.]

### News from the Nursing World.

### THE CHILDREN'S STORY OF THE QUEEN'S REIGN.

Through the "Children's Salon" it is proposed to raise contributions towards the Jubilee Nurses' Commemoration Fund, the children undertaking collecting-cards for the purpose. Miss Northcroft gave a lecture, entitled "The Children's Story of the Queen's Reign," on behalf of this children's tribute to the fund, at Eastbourne last week, when a large audience gathered in the Mayor's parlour to hear her, and many cards were distributed by the young members of the "Salon." On Monday the Duke of Westminster gave the use of Grosvenor House for a lecture on the same subject, by Mrs. Jack Johnson.

### A ROYAL LECTURER.

It is reported that the Queen of Portugal has been putting to very practical use the knowledge of anatomy gained in the course of her medical studies. She has been experimenting with X-rays on the ladies of the Court, and giving illustrated lectures on the evil results of tight-lacing, the pictures aiding Her Majesty's arguments with remorseless truthfulness of details.

### LECTURES ON NURSING.

A course of seven lectures on "Sick Nursing" has just been given by Nurse Amy Smith, of the West Riding Nurses' Association, Halifax, at Farsley. The lectures have been very largely attended, and much appreciated, and at the end of the course Nurse Smith was presented by the Vicar, on behalf of the audience, with a morocco writing case. The Mayoress of Halifax was present, and said a few words on the value of trained nurses, and of a knowledge of sanitation and nursing to women generally.

### NURSING AT POPLAR.

At the annual meeting at the Poplar Hospital for Accidents last week, the Hon. Sydney Holland, in commenting upon the conscientious and thorough manner in which the nursing department is managed by Miss Bland, remarked that the nursing "got better every year, and there was nothing in which he took greater pleasure than in the belief that it was second to none." The reason may possibly be gathered from the following paragraph in the printed report, which speaks for itself: "We have tried to make the conditions of service for our nurses fair and reasonable, and to study their comfort. Although no bargain is suggested, and it would spoil the spirit of all hospital work if it were even thought of, we believe that we are, as a fact, repaid over and over again by having a loyal and devoted staff, and we think we may add, a happy one; certainly our nurses are better in health for the longer time off duty which they now have. It is a miserable policy to run a hospital at the expense of its nurses. We believe that the patients directly benefit almost as much as the nurses themselves, by giving nurses every week the opportunity of a full half-day's rest, and every month 60 hours right away from the wards." It is proposed, as soon as possible, to build rooms for the sisters off their wards. Speaking of this at the meeting Mr. Holland admitted that when the hospital was built he did not know as much of nurses as he did now, and he then thought if they had ward sitting-rooms they would be more often there than in the wards. He had learnt now that the difficulty was to keep the sisters away from their wards!

### TRAINING FOR MIDWIVES.

In consequence of the increasing demand for country district midwives, the council of the Midwives' Institute have decided to start a very thorough course of teaching for those who wish to be prepared for the L.O.S. examination, and "to receive such detailed instruction as shall aid them in their future sphere, whether their work is to be that of a midwife or of a district monthly nurse under a medical man." The course will begin on Monday, April 26th, and finish in time for the July obstetrical examination. There will be eighteen lectures by T. R. Humphreys, Esq., L.R.C.P.; lectures on monthly nursing by Mrs. Hewer, a trained nurse and midwife; and on sanitation by Miss Lamport, a trained nurse and associate of the Sanitary Institute, &c. Classes will be held by a midwife experienced in teaching, and demonstrations in bed-making, bandaging, poultice-making, &c., given, while arrangements have been made with midwife members to reserve vacancies for pupils recommended through the Institute. Full particulars will be found in the April number of *Nursing Notes*. Intending pupils should at once apply to the Secretary of the Institute, 12, Buckingham Street, Strand.

### SAD DEATH OF A NURSE.

A sad occurrence is reported from the Windsor Royal Infirmary. One of the charge nurses, who was having some teeth extracted under gas and ether, collapsed suddenly, and, in spite of every effort to save her, died without recovering consciousness. Nurse Gath was a north country woman, and had been but a short time at Windsor.

### ABERDEEN DISTRICT NURSING ASSOCIATION.

A superintendent and seven Queen's nurses are at work in Aberdeen, a staff none too large for the heavy work of several very scattered districts. It is, by the way, something of a reproach to Aberdeen that the committee should have to report that "it has been found impossible to make any special arrangements as to fares" (tram fares) "for the nurses, such as exist in Dundee and some other towns." Considering the constant use made by the nurses of these trams in the course of their journeyings, the companies should surely be proud to aid their work amongst the poor by making some reduction on their tickets. The proceeds of a bazaar last year have been used in repairing and furnishing a pleasant and cheerful home for the nurses. The association finds its ordinary annual income insufficient for the work to be done, and the committee plead for additional subscriptions, an increased income of £140 to £150 being urgently required.

### NURSES ON THE GOLD COAST.

The English nurses who have gone out to take charge of the Government hospital at Accra, Miss Hanley as matron, and Miss A. M. Deeks as senior sister, met with a very friendly reception on their arrival last month. The few resident European ladies made the new comers very welcome, and they were promptly presented to the Governor, at his reception, by the wife of the Colonial Secretary, Mrs. Hodgson. The hospital at Accra is prettily situated near the sea, and contains some sixty beds, one part of the building being portioned off for Europeans; there is also a special ward for Houssas, the native troops. A pleasant, well-furnished bungalow is allotted to Miss Hanley and her staff. On the voyage out a three days' stay was made at Sierra Leone, where visits were exchanged with the sisters at Princess Christian's Hospital.

### NURSING IN AUSTRALIA.

A proof of the excellent reputation of the training at the Prince Alfred Hospital, Sydney, is to be found in the fact that the nurses of that training school obtain the majority of the matronships at other Australian hospitals when vacancies occur. Miss Pope, one of Miss McGahey's "sisters" has just been appointed matron to the Rydalmere Hospital for the Insane, an important post, for which she possesses excellent qualifications. Miss McGahey takes infinite and personal trouble to secure that her nurses shall be fitted for the responsibilities of such appointments, and herself instructs those who are thinking of applying for matronships in housekeeping, book-keeping, and the many details of general hospital management which do not come within the ordinary scope of the staff nurse or ward sister.

### KETTERING DISTRICT NURSING ASSOCIATION.

The Kettering District Nursing Association, affiliated to the Queen's Jubilee Institute, has a very satisfactory report to make of the past year's work. The work of the nurses has been well supported in Kettering. Nurse Lloyd and Nurse Milnes were cordially thanked at the annual meeting for "the ability and energy with which they carried out their duties." "The association," said Mr. E. P. Toller, "was to be congratulated on all its officers, but the foundation of all their operations rested almost entirely upon their nurses; they were particularly fortunate in having such excellent, enthusiastic, and able nurses." A useful gift during the year was that of a bicycle for the nurses, which had proved of great service.

### EIGHTEEN HOURS ON DUTY.

The Skipton Board of Guardians are not likely to retain any conscientious nurses in their service until they have taken some steps to improve the conditions under which they have to work. On the resignation of the last two nurses, the state of affairs was commented on by Father Sharp, who pointed out that there were two nurses taking day and night duty in shifts of 12 hours each, once a week taking "18 hours each without a single break. That," he said, "seemed to him to be downright cruelty; in addition to which, there was not even a room to sit in or a comfortable place in which to take a meal. The place was no hospital at all; and if anything did happen, the Board would certainly be shown up to some tune. Eighteen hours' work, with the approval of the Board, was outrageous; and even 12 hours a day was much too long with 18 or 19 very bad cases. He would suggest the whole matter be taken into consideration, and some alteration brought about in the conditions under which the nurses performed their work. Perhaps it would be feasible to appoint a probationer." Another Guardian added that "the physical power required from their nurses would tax the strongest Guardian"; and a resolution was agreed to referring the matter to the House Committee for early consideration, with a view to some assistance being given the nurses in their work.

### DISTRICT NURSING IN SOUTH WALES.

A proposal has been made to provide a district nurse for Neyland (South Wales), and a largely attended meeting was recently held to consider the matter. Some wonderful reasons have been advanced in the local press against this very desirable step, which show clearly that it is quite time the educative influence of a district nurse made itself felt, not only among the "very poor" of the neighbourhood in question, but amongst other folk who ought to know better. A critic remarks that "the very poor are mostly the very ignorant, and they for whom the nurse would be intended are the ones most likely to refuse her assistance." Though this writer kindly adds that he has "nothing to say in a general way against the institution of district nurses," he is nevertheless of the opinion, one which died a natural death long ago amongst most educated people, "that in such cases the presence of a lady in the cottage would tend to do more harm than good by the worry she would cause to the poor patient!" Our friend has evidently no acquaintance with district nurses and their work.

### SHORT ITEMS.

The Committee of the St. John Ambulance Association has received the sanction of His Royal Highness the Prince of Wales to form an ambulance station at the Victorian Era Exhibition, and to keep it provided with attendants who will render first-aid in case of accidents.—The special committee appointed to conside a scheme for extending and maintaining the work of the Queen's Jubilee Nurses in Northampton have unanimously decided that the idea cannot be satisfactorily carried out.—Amongst other approved schemes for the Queen's Commemoration in Birmingham, it has been decided to grant £2,500 to the District Nursing Society for the establishment of two homes, to be called the "Queen Victoria Commemoration Homes."—It was announced, at the recent annual meeting of the Gloucester District Nursing Society, that the committee had decided to affiliate with the Queen's Jubilee Institute, "and thus secure the permanent efficiency of the institution."—The Stockton and Thornaby District Nursing Association received in workmen's subscriptions during 1896 £475 3s. 4d., an increase of £34 17s. 4d. as compared with the previous year.—Forty-three midwives and one hundred and fifty-eight nurse pupils were admitted for training at the City of London Lying-in Hospital during the past year. Of the forty-three midwives, thirty-eight presented themselves for the L.O.S. examination, and all passed.

### Lectures to Surgical Nurses.

By H. A. Latimer, M.D. (Dunelm), M.R.C.S. of Eng., L.S.A. of London, Consulting Surgeon, Swansea Hospital; President of the Swansea Medical Society; Lecturer and Examiner of the St. John Ambulance Association, &c.

### IV.—THE HEART AND BLOOD-VESSELS—MODE OF CIRCULATION OF THE BLOOD—NATURE'S METHOD OF ARRESTING HÆMORRHAGE.

The heart is a somewhat pear-shaped body, being larger at its base above than at its apex, which is below. Vertical and horizontal walls divide it into two chambers on each side, right and left, the two upper chambers, which receive blood from veins, being called auricles, and the two lower chambers, from whence blood is expelled through arteries, being called ventricles. By successive acts of opening and shutting of these chambers blood is received and passed through them, the current of circulation being directed, when the organ is sound in its structure, by the opening of apertures to allow the blood to pass through them, whilst its return in the wrong direction is prevented taking place by the shutting back of flaps or "valves," which spring into place and close the opening when the wave of fluid has passed them. I purposely refrain from going into greater details of anatomy and physiology than the above, as I know that you have received instruction on these points by other lecturers, and because it is hardly necessary for me to do so, as you will not be concerned at any time, as surgical nurses, with the practical matters of bleeding from the heart. It is different, however, with the vessels which receive and carry blood when it is in process of circulation through the body; for you will see, and will often have to deal with its escape from these vessels, and with the constitutional results of the same, following such an escape. Let us then see in what manner the blood circulates: through what vessels it passes: and how we must deal with hæmorrhage, should such occur. Taking our starting point with the purified blood as it has been expelled from the left ventricle by a sudden contraction of that chamber, we find a wave of it is received by the first of the arteries; this artery, as do all the later continuation of arteries, expands on receiving the wave, and, being made of elastic material, contracts again when the wave has passed on. It is this expansion and jerk which you feel when you examine the pulse at the wrist. Everywhere the arteries, which are carrying purified blood of a bright scarlet colour, expand and contract in this way; and, as a further means of pushing on the flow of blood, as they become small in size they have a circular lining of muscular fibres by which they are enabled to be expanded or contracted in response to orders sent them through sympathetic nerves. When the arteries have diminished, and have become very small, the channel of blood-flow is continued through vessels of so minute a size that they are called capillaries (from the Latin word *capillus*, a hair), and it is whilst flowing through these capillaries that nutriment to the tissues is given out where required, that oxygen is liberated, and that effete materials and carbonic acid gas, which are the results of the wear and tear of the body, are absorbed, and are carried away in the blood-stream, to be got rid of by the various organs whose function it is to expel them from the system. The blood which is now impure is no longer bright scarlet in colour. It has hitherto been flowing away from the heart to the head, the fingers, and the toes; and the vessels in which it has been travelling have been getting smaller and smaller as they have divided and subdivided. It has now reached the veins, which, of small size at first, become larger and larger as they make their way from the extremities of the body to the heart, and it is of a dark purple hue. It is no longer pushed in jerks, but flows in a more or less steady stream, being made to move by the pressure of the column of blood which is behind it, and by a mechanical arrangement of valves within the veins, which fold up against their walls to allow of the blood passing in the desired direction, but flap back to oppose its returning, should anything be stopping it further on. From what I have said, you will see that you can tell from what kind of vessel blood is issuing by its colour, the way in which it flows, and the effect which is produced by pressure which you are making to stop the bleeding. If an artery has been cut the blood will come from it in jerks; its colour will be bright scarlet, and proper pressure applied between the heart and the wound will stop the hæmorrhage. If capillaries have been severed the blood will simply ooze out. If veins have been opened the blood will well out in a steady, continuous stream; it will be purple, and pressure applied on the further side of the wound will stop it.

I need not tell you what serious consequences result from the loss of much blood. The results may be immediate or remote. At the time it occurs we may have fainting, or collapse; and if the patient rallies from these conditions, he may be left weak and delicate for a long time to come. It is very important, then, to know what Nature does for arresting hæmorrhage, and what surgeons and nurses can do to the same end.

When, from fright, emotion, or weakness, fainting takes place (a condition in which the heart beats so feebly that the brain does not receive a sufficient supply of blood to stimulate it to do its work), the blood flows so languidly through its vessels that it ceases to make its escape from a wound, to any great extent; and another thing which always takes place when blood is stagnant now occurs—a clot is formed at the wound, and for some little distance beyond it, and this clot seals up the opening and so stops the bleeding. Moreover, if an artery has been bruised or crushed whilst torn across, the muscles I spoke of just now as existing within its walls contract, and so close it. It is by this provision of Nature that animals are enabled to gnaw off their injured limbs without suffering from loss of blood; and in cases when limbs have been crushed, whilst being torn off by machinery, you will often see patients admitted to the hospital with formidable wounds which are not bleeding at all, for the same reason. Surgeons often make use of this property of contraction possessed by arteries in a way which I shall presently tell you of, as I have now reached the point when I have to tell you what measures they take for guarding against hæmorrhage and for controlling it, when it has taken place, and what your duties are under like circumstances.

Let us deal with this question from the point of prevention and cure. Surgeons have to guard against bleeding when they perform operations, for they cannot remove limbs and tumours and do other surgical acts without cutting through blood-vessels of one kind or another; and both surgeons and nurses have to attend to cases when sudden bleeding sets in, and has to be dealt with promptly. In the case of the operation measures are taken to prevent the bleeding occurring.

These measures I propose to deal with in my next lecture, so I will postpone their consideration for the present.

### Post-Graduate Clinics for Nurses.

By a Trained Nurse.

VIII.—THE PERSONNEL OF THE NURSE (*continued*).

Now, my ideal nurse is a particularly nice, healthy human woman, not an ideal on paper, dressed in a soft-coloured gown, boasting little starch, with no aggressive weapons of surgery depending from her belt, possessed of gifts of perception, patience, and sympathy. It is hardly necessary to insist that she must be cheerful, for Nature seems to have endowed the whole race of nurses with this most enviable quality. I would like her to be a woman of authority, but to assume the reins of office by degrees and by gradual ascent. In entering the sick-room it is right that she should have a latent intention of putting everything in order, of altering any existing unhygienic surroundings of her patient, and of making his conditions the best possible for him as an individual, and for him as a "case." But in private practice it is essential to begin with the thin edge of the wedge, and to quietly and gradually, day by day, put wrong things right and introduce reforms with wariness and tact. The first week of attendance on a sick person is, perhaps, the most trying period, for during this time the nurse is more or less of an experiment—on probation—for she is a stranger whose authority must be justified, and whose kindness must be assured. In that first week it will be necessary to measure strength with the patient's friends—but by measuring strength, I do not mean measuring swords. It consists simply in proving herself to be a friend of the patient, and a friend of the family. And the manner in which such proof can be given is learnt more from sympathy and feeling than by the exercise of brain faculty.

Speaking, generally, before the advent of the trained nurse there has been a period, more or less prolonged, of home nursing, various members of the family trying their 'prentice hands on the patient, and they will probably have deviated considerably from the straight path of nursing as understood by the trained nurse. One of the daughters or sisters will probably have attended a course of ambulance and nursing lectures, and will thus have acquired a smattering of the essentials of ventilation, and the necessity for keeping a sick bed free from crumbs! But the nurse will usually find that the nursing, so far as it has gone, has been done in more or less scrappy fashion—kindly, and with the best motives, but without plan or method. Her first difficulty then will be to establish some system of regularity, not only in the patient's *regimen*, but in the clockwork of the sick room, which can only run smoothly by a discreet punctuality, tempered, be it remembered, to suit the needs of a single person. In a ward each patient must be regarded more or less as a factor fitting in to the mechanism of the whole ward. The private patient, on the other hand, *is* the mechanism with whom all his friends and relations—and even the nurse—must fit in and accord, and this point of view makes all the difference. In Hospital, where there are 20 patients to be washed, dressed, and breakfasted by a certain hour, the very sickest among them, though left to the last, have often to be disturbed at a cruelly early hour, when a prolonged morning sleep would be of the utmost value to them, for, where numbers are gathered together, there arises an absolute necessity to keep regular routine and order. In private practice this necessity does not exist, and to establish early morning hours in the sick room is to indulge in very bad nursing habits. It frequently happens when two nurses are engaged on "a case" they strive to map out the hours usual in hospital for day and night duty and "time off," and frequently the attempt works badly and uncomfortably. The average patient—the Londoner, at any rate—is not accustomed to be "washed and breakfasted" by nine a.m., and not only he, but the household, will usually find such a system uncomfortable. And for the sake of the patient it is well to make no hard and fast rule as to the hour at which his "room should be ready." The room at all times—even in the middle of the night—should be in such a condition that the nurse could admit without a tremor the whole College of Physicians, and a patient must not be disturbed from his restorative morning sleep—Nature's remedy for a restless, bad night—by any consideration of time. The surroundings must be subordinated to the sick, not the sick to the surroundings or to a doctor's visit. To some ears it will probably sound like heresy to state that everything must not be sacrificed to the doctor's visit, for that this should be so is the beginning, middle, and end of many a nurse's creed. And so far as her relation to the doctor goes it is absolutely true. But no doctor exacts or would for one moment sanction, did he know it, the long and wearisome process of tidying up, furbishing, and fidgeting of the patient which so commonly begins some two hours before "the doctor comes." The exhaustion and low vitality exhibited by a patient who is ill enough to receive a nine o'clock visit from his doctor is far too often the result of a strain to "keep up appearances" which the medical attendant would be the first to repudiate as being due or necessary to him in his professional position. The standard of preparation is raised too high, and the fatigue consequent on all such fuss is decidedly injurious to the really ill. To some convalescents it is an amusement, but even amusement must not be overdone. A common plan in New York, which works out most satisfactorily—and I speak from tried and personal experience—is to arrange when two nurses are engaged on a private case that one shall begin her duty at four a.m., leaving off at four p.m. The second nurse then relieves her till the following four a.m. By this means—and the system has been introduced into the Royal Infirmary at Glasgow—each nurse gets a fair share of daylight, and the duties are equally divided, one doing the morning toilet and day preparation; while the other prepares and settles her patient for the night. There is no difficulty whatever in arranging the meals if materials be at hand for furnishing a breakfast to the retiring nurse before she "goes off duty," which the oncoming nurse may share. The one who has taken night duty will be up and dressed for the mid-day luncheon, after which she can take her constitutional and social recreation. This arrangement does not hold good if the nature of the illness call for the assistance of two nurses for the bed making and washing. Then, the older fashion of a day and night nurse is preferable, so that two may be on duty for a short time in the morning and at bed-time. But the plan works well in ordinary cases. The nurses find it infinitely better from a health point of view, and it makes no difference to the patient, for no disturbance or noise need be effected by a change of nurse in the early morning hours. Where space is limited and the two nurses must perforce occupy the same bed-room, the nurse who retires at four a.m. gets up probably at one p.m., while the nurse due to go off at four p.m. probably does not go to bed till seven p.m., so that the bed-room has six consecutive hours in which to air. In all cases where two nurses occupy the same room two beds should be provided, and this should not occasion inconvenience, for most households possess a small folding bedstead, so that the necessary comfort of a bed "of one's own" can easily be requisitioned.

### Hospital Nursing: Where and How to Train.

WHEN the would-be nurse has firmly made up her mind that nursing is her vocation, if she has no special leanings towards some particular training school she begins to wonder at which hospital to apply for a vacancy as probationer, and to look for definite information as to the terms upon which that training is to be secured. She wishes to know for how long she will be required to bind herself to the service of the institution where she receives her training; whether she will have to pay, or will be paid; if she has to provide her own uniform, or if it is supplied by the hospital; and which of the many institutions available will best suit her circumstances and inclinations—always supposing she is fortunate enough to become a "selected candidate." It will, perhaps, be helpful to those who are contemplating a nurse's training if we sum up very briefly the terms under which it is to be obtained at the chief London and provincial hospitals. Taking the general hospitals in London first, in the order of number of beds:—

At the LONDON HOSPITAL, Whitechapel Road, E. (800 beds), non-paying probationers must be between 25 and 35 years of age, paying probationers between 22 and 40. The former enter on a three years' engagement, two years' training and a third year in the service of the hospital, certificates being given at the end of the second year. They receive a salary, uniform, and an allowance for washing. Paying probationers are received for periods of three months for a fee of 13 guineas, and may be transferred to the regular staff if they wish and prove suitable. Miss Lückes is the matron.

At ST. BARTHOLOMEW'S HOSPITAL, Smithfield, E.C. (744 beds), probationers are required to sign an agreement for four years—three for training and one on the staff. Their age must be not less than 23 nor more than 35. They are paid a salary, and are provided by the hospital with a certain amount of uniform and washing. Before entering the hospital probationers are required to pass a preliminary examination. Paying probationers are received for periods of not less than three months, at a payment of 13 guineas. Age between 22 and 40. Miss Isla Stewart is the matron.

Probationers are received at GUY'S HOSPITAL, St. Thomas's Street, S.E. (695 beds), for three years' training, during which time they are paid a salary, and provided with uniform and washing. They are eligible for promotion as ward sisters. Lady pupils are received for one year's training, paying 13 guineas a quarter, providing their own uniform and paying their laundry expenses. Lady pupils are eligible to become sisters of wards. The age for probationers is between 23 and 32, for lady pupils between 24 and 32. Miss Nott-Bower is the matron.

Probationers entering the NIGHTINGALE TRAINING SCHOOL, St. Thomas's Hospital, S.E. (569 beds), sign an agreement to remain in its service for four years, receiving salary, uniform, and laundry allowance. They are not eligible for promotion as ward sisters. Special probationers, who must be "gentlewomen," between the ages of 25 and 33, either pay £30 and sign an agreement for three years, or £52 and sign for two years. Special probationers may be promoted to the post of ward sister. Miss Gordon is the matron.

At ST. GEORGE'S HOSPITAL, Hyde Park Corner (351 beds), probationers are taken for a term of three years, being promoted, if satisfactory, to the position of staff nurse at the end of the second year. They receive a salary, uniform, and washing allowance. Mrs. Coster is the lady superintendent.

Non-paying probationers at the MIDDLESEX HOSPITAL, W. (321 beds) are chiefly drawn from the domestic servant class. They are appointed staff nurses, if suitable, at the end of a year's training. There are a few lady probationers, who pay one guinea weekly for six or twelve months' training. Certificates are given on leaving the hospital. The non-paying probationers, in nddition to a salary, are provided with uniform, and the washing of their caps, cuffs, collars, and aprons is undertaken by the hospital. Miss Thorold is the lady superintendent.

Three years' training is given at ST. MARY'S HOSPITAL, Paddington (281 beds). Candidates must be between 23 and 35 years of age. The certificate is given at the end of the three years. They receive a salary, indoor uniform, and a certain amount of washing. Paying probationers are admitted for one year's training, the fees ranging from £30 for this period, to £50, if a separate bedroom is desired. They provide their own uniform and pay for their own washing. Miss Medill is the matron.

Probationer-nurses and special probationers are received for training at KING'S COLLEGE HOSPITAL, Lincoln's Inn Fields, W.C. (220 beds). Applicants to be between 25 and 30 years of age. The term of training is three years, probationer-nurses being unpaid for the first year, while special probationers pay ten guineas a quarter for the first year, five guineas a quarter for the second, and, if approved by the committee, are appointed unpaid staff nurse during the third. Probationer-nurses receive a salary for the second and subsequent years. Indoor and outdoor uniform is supplied by the hospital to all probationers and nurses, and an allowance is made weekly for washing. Miss Monk is the sister-matron.

UNIVERSITY COLLEGE HOSPITAL, Gower Street, W.C. (208 beds), is nursed by the community of All Saints'. Probationers are admitted for training between the ages of 24 and 30, and, except in a few exceptional instances, pay for their training. Payment is for the first year £30, exclusive of washing, and those who wish to qualify for a certificate, only given at the end of three years, pay £20 for the second, and £15 for the third year. During the first six months probationers pay for their uniform; afterwards this is provided by the hospital, and the washing of the uniform. Sister Cecilia is the sister superior.

At CHARING CROSS HOSPITAL, Agar Street, Strand (175 beds), probationers are received for training between the ages of 23 and 34, on a three years' engagement, one year's probation, and two additional years in the service of the hospital. They are unpaid for the first twelve months, receiving uniform and washing; and are paid a salary for the subsequent years. Special probationers are admitted for twelve months' training, for which the fee is 52 guineas, the age considered desirable being from 23 to 35. Miss H. A. C. Gordon is the lady superintendent.

The ROYAL FREE HOSPITAL, Gray's Inn Road, W.C. (160 beds), requires non-paying probationers to sign a four years' engagement, candidates being eligible between the ages of 23 and 35. No salary is given for the first year. Probationers enter on a three months' trial, during which time they provide their own uniform; afterwards a proportion of the uniform is provided by the hospital. Paying probationers are admitted for periods of six months, paying thirteen guineas a quarter, and providing their own uniform, and paying for their washing. They must be between the ages of 23 and 45. Miss Wedgwood is the matron.

At the GREAT NORTHERN CENTRAL HOSPITAL, Holloway Road, N. (155 beds), probationers are received for three years' training, between the ages of 23 and 35. They pay a premium of £10, receiving a salary for the second and third years. Uniform is supplied by the hospital, and an allowance made for laundry expenses. Miss Hull is the matron.

At the LONDON TEMPERANCE HOSPITAL, Hampstead Road, N.W., probationers are received on a three years' engagement, for which they are required to pay a fee of 30 guineas for the first year, receiving a salary for the remaining two years, or for a period of one year, during which they pay at the rate of one guinea weekly. The age considered desirable is 23 to 30. Uniform has to be provided by the probationers, and a weekly charge of 1s. 6d. is made for washing during the first year. Miss Orme is the matron.

### How to Become a Dispenser.

DISPENSING as an occupation for women is becoming very popular, and many inquiries are addressed to us respecting the training required, the length of time such training must occupy, and how would-be dispensers should set about studying pharmacy with a view to making a livelihood as a dispenser.

Other inquirers there are to whom a word of warning should be addressed. Many nurses seem to be possessed with the idea that "a little knowledge of dispensing" will be useful to them, and that they can "pick up" enough of such knowledge in odd half-hours, and by the study of a text-book or two. But it is a very serious matter for unqualified people to meddle with drugs, and no nurse, or anyone else for that matter, should attempt to do so until she has at least passed the assistants' examination of the Apothecaries' Hall. By that time she will have learnt enough to make her something more than an active danger to the public, which the dabbler in such a life and death matter as the compounding of medicines cannot fail to be.

To any woman wishing to train as a dispenser, we would recommend a small pamphlet just published by Miss Bradbury, resident dispenser at the Ryde Dispensary, in which she embodies the results of her own experience. This pamphlet can be obtained from F. W. Flux and Co., 12, John Street, Ryde, and its price is one shilling. Miss Bradbury's advice to those who wish to take up dispensing as a profession is in the first place to obtain the certificate of the Apothecaries' Hall to act as an assistant, for which she considers six to eight months' steady daily work should suffice "for a girl with ordinary capabilities, and giving up all her time to it." A year's work would be better. This examination is held six times a year, and the fee is two guineas. This much achieved, an appointment can be obtained, and the three years' practical work which is required from candidates presenting themselves for the higher examinations of the Pharmaceutical Society entered upon. Thus, as the Apothecaries' Hall certificate is a sufficient qualification for many appointments, the necessary experience for the higher examinations can be gained while the learner is at the same time earning her living.

Then comes the question, where is the needful practical and theoretical training for the first examination to be had? There are, of course, schools of pharmacy open to women, but the best method is to become a pupil at some hospital or dispensary, where the practical knowledge gained will be of special and great value to those who hope in time to have the management of similar institutions. There is not much difficulty now in obtaining instruction in this way, for many ladies who hold appointments as dispensers at hospitals or dispensaries undertake the training of pupils. Ladies hold these appointments at several hospitals; in London amongst others at the New Hospital for Women, Euston Road, N., at the London Temperance Hospital, Hampstead Road, N., and at the Royal Eye Hospital, Southwark; there is a lady dispenser at the Ventnor Hospital, while Miss Bradbury has taken pupils at the Ryde Dispensary for some years.

The first step, therefore, to be taken is to set about getting the necessary instruction for passing the Apothecaries' Hall Examination, and application should be made to a qualified dispenser on this point. With regard to the books for study, we may quote those recommended in Miss Bradbury's pamphlet. These are "The British Pharmacopœia," Squire's "Comparison to the British Pharmacopœia," Martindale's "Extra Pharmacopœia," Attfield's "Chemistry," Remsen's "Elements of Chemistry," "Materia Medica and Therapeutics," by Mitchell Bruce; Will's "Organic Materia Medica," "Elements of Pharmacy, Materia Medica, and Therapeutics," Whilta; Bentley's "Botany," and Ince's "Latin Grammar of Pharmacy."

The year or six months' study means daily practice as well as theoretical work. The Apothecaries Assistants' Examination passed, and a post obtained, the Minor Examination of the Pharmaceutical Society can then be worked up for at leisure. Particulars respecting these examinations can be obtained from the Secretary of the Apothecaries Hall Society, Blackfriars, E.C.; and from the Secretary of the Pharmaceutical Society, 17, Bloomsbury Square, W.C.

### Where to Go.

THE VICTORIA COMMEMORATION CLUB.—Dr. Ewen Y. Maclean gave the first of his interesting and important series of lectures on the "Nursing of Gynecological Operations" in the class-room of the Victoria (Commemoration) Club on the 30th inst. The next lecture will take place on Tuesday, April 6th, at three p.m., the subject to be "The Use and Abuse of Antiseptics."—Miss Earle, who has a particularly charming and lucid method, holds invalid cookery demonstrations in the class-room of the club on Tuesday evenings at seven p.m. during the month of April. The price of a single admission to both lectures and demonstrations is 1s. to members and 1s. 6d. to non-members. —Members wishing to stay in London can secure bedrooms at 3s. a night, in close proximity to the club, by applying to the Secretary.

ST. GEORGE'S HALL, LANGHAM PLACE, W.—A grand variety matinée will be given at this hall on Saturday, April 10th, at three o'clock, in aid of the Boys' Surgical Home, Banstead. The entertainment is given under the immediate patronage of the Empress Frederick. Tickets, 7s. 6d. and 5s., may be obtained at Mitchell's Library, Old Bond Street, or from Mrs. W. Lawrence Smith, 18A, Collingham Gardens, South Kensington.

ROYAL BRITISH NURSES' ASSOCIATION.—The quarterly meeting of the General Council of the Royal British Nurses' Association will be held at 17, Old Cavendish Street, W., on Friday, April 9th, at five p.m. The fifth sessional lecture of the season will be given on Friday, April 30th, at eight p.m., at the offices of the Royal British Nurses' Association, 17, Old Cavendish Street, by Miss G. Scott (late matron of the Sussex County Hospital), the subject being "The Failures and Successes of Private Nurses." Members are admitted free, the general public on payment of 1s.

### Death in our Ranks.

WE regret to announce the death of Nurse Laurie Paterson, which took place last week. Nurse Paterson, after nearly completing her three years' training at the Fir Vale Infirmary, Sheffield, was obliged to leave in consequence of ill-health following influenza. Her death has been much felt among her fellow nurses, by whom she was much beloved. The funeral took place last Friday at Wellington, Shrewsbury. Mrs. Lawson, the Lady Superintendent of Fir Vale, and the nursing staff, sent a harp of beautiful white flowers as a last tribute of affection and respect "from sorrowful fellow-workers."

### Minor Appointments.

DONCASTER UNION INFIRMARY.—Miss Annie Rodwell has been appointed Charge Nurse at this infirmary. She was trained at the Chorlton Workhouse Union Infirmary.

CORPORATION FEVER HOSPITAL, BOOTLE.—Miss E. Rudd has been appointed Sister at this institution. Miss Rudd was trained at the Glasgow Royal Infirmary, has gained experience in fever nursing at the Hull Sanatorium, and has also had six years' experience in district work. She has very good testimonials.

### Feeding the Invalid.

It is a recognised fact in the present day that diet has much to do in hastening or retarding convalescence. At many of the best training schools cooking forms part of the nursing curriculum, and thus the value of the private nurse is enhanced. But there are still many institutions where nurses receive no initiation into the art of cookery, and many nurses whose training ended before the true value of knowledge in this direction was realised. It will be our endeavour in these columns to assist by suggestion those nurses who are already expert cooks, and to offer to others a diet which shall be suitable in convalescence and under certain conditions of disease, such as typhoid and diabetes. It may not be out of place to insist on the importance of an appetising manner of serving all meals, for the appearance of the tray when it is brought to the invalid has much to do with the subsequent enjoyment or refusal of a meal. Miss Hampton, in her book on "Nursing," devotes a most useful chapter to this subject, which might be read with advantage by all tending the sick.

Cloths should be spotless, glass and silver as bright as possible, and a small quantity of food only be put before a patient. The following ménu suggests a variety for a convalescent recovering from an ordinary illness, and the selection has been made of such dishes which could follow each other as the patient increases in strength.

Sunday.

Breakfast.—Coddled eggs, toast and butter, hot milk.
Dinner.—Filleted sole, sago soufflé.
Tea.—Marmalade sandwiches, Vienna bread and butter, tea.

Monday.

Breakfast.—Whiting, brown bread and butter, coffee.
Dinner.—Sweetbread, custard pudding.
Tea.—Buttered toast, sponge cake, tea.

Tuesday.

Breakfast.—Poached egg, toast and butter, tea.
Dinner.—Oyster soup, roast chicken, bread sauce.
Tea.—Tea rusks, brown bread and butter, chocolate.

Wednesday.

Breakfast.—Fried smelts, bread and butter, coffee, fruit.
Dinner.—Minced sweetbread and chicken, tapioca soufflé.
Tea.—Potted meat sandwiches, angel cake, tea.

Thursday.

Breakfast.—Scrambled egg, toast and butter, tea, fruit.
Dinner.—Noisettes of mutton, mashed potatoes, lemon jelly.
Tea.—Chocolate, bread and butter, French lettuce, apricot sandwiches.

Friday.

Breakfast.—Scolloped fish, bread and butter, fruit, coffee.
Dinner.—Roast pigeon, bread sauce, potato chips, apple snow.
Tea.—Hot tea cake, egg sandwiches, tea.

Saturday.

Breakfast.—Soufflé omelette, bread and butter, hot milk, fruit.
Dinner.—Fillets of beef, mashed potatoes, lemon pudding.
Tea.—Hovis bread and butter, honey sandwiches, tea.

Recipe for Fillets of Sole.

Remove the fillets from a medium-sized fresh sole by passing a knife down the centre of the fish, and work the fillet off by keeping the knife close to the bone. Lay the fillets on a wet board, and bat them with a heavy knife that has been dipped in cold water. Trim them, and season with a little salt and a few drops of lemon juice, fold the fillet over and trim the pointed end, lay them on a buttered baking tin, and pour one tablespoonful of cream over each fillet and one in the tin. Cover them with a buttered paper, and place the tin inside another, the under one having boiling water in it. Place both tins in a moderate oven for twenty minutes, occasionally basting the fillets. Serve on a small hot dish, and sprinkle with chopped parsley.

Any recipes can be had by addressing and enclosing seven stamps to "Invalid Cookery," The Hospital Office, 28 and 29, Southampton Street, Strand, W.C.

### Everybody's Opinion.

[Correspondence on all subjects is invited, but we cannot in any way be responsible for the opinions expressed by our correspondents. No communication can be entertained if the name and address of the correspondent is not given, or unless one side of the paper only is written on.]

NURSES' EARNINGS.

"A Correspondent" writes: Can any reader of The Hospital suggest a reason why a nurse should receive such a much lower salary than high-class cooks, ladies' maids, type writers, &c. These people often receive from £50 to £100 per annum, and all found. A nurse, and especially a district nurse, has far harder work, often a rough time of it battling with the elements in country districts, and on the roll of the "Queen's" seldom receives more than £30—at the outside £35. The writer, a nurse of some years' standing, has lately had the matter thrust home very forcibly, and is altogether saddened and depressed at the low salaries nurses as a class receive. Would it not be a good way of celebrating the Queen's long reign to increase the salaries of "Queen's" nurses, and to pay half the premiums of those nurses who belong to the Pension Fund from the money being collected to augment the capital of the Jubilee Fund? I enclose my card as reference.

### Novelties for Nurses.

MESSRS. SOUTHALL BROS.' SPECIALITIES.

To this firm we are indebted for many valuable contributions to the sick room in the shape of absorbent dressings. They have now succeeded in bringing out a halfpenny "sanitary towel," the importance of which cannot be over-estimated. It is composed of a mixture of finely divided cellulose, with a pure white sufusorial earth called "Dimatos." Dimatos is a soft, inert powder, lighter than magnesia, and capable of absorbing six times its weight of liquids. This powder is completely incorporated with the cellulose, and forms the nucleus of the pad. It is then enveloped in absorbent wool, and the whole enclosed in a covering of gauze. To women of the working classes the advantages of being able to obtain a packet of these articles at half the price that washing would cost is incalculable, and the more widely they are made known the greater demand we confidently predict for them. Intending purchasers will be well advised to send for size O "sanitary towel," price 6d. per packet.

### Presentations.

On Thursday, 16th inst., at St. Saviour's Infirmary, East Dulwich, H. Elwin Harris, Esq., B.A., M.B., F.R.C.S.(Eng.), the medical superintendent, was presented by the staff of the above institution with a handsome gold watch and chain, suitably inscribed, on the occasion of his resigning his appointment to enter into private practice. The Rev. G. Elliott, in presenting the testimonial on behalf and in the presence of the whole of the staff of the infirmary, spoke in warm terms of the many improvements which Dr. Harris had instituted during his five and a half years' tenure of office, laying special stress on his organisation and the method in which the nurses' training school was conducted, and also his championship of the nurses' cause in the recent discussion on the Poor Law Superannuation Act. Dr. Harris, in his reply, thanked the staff for their very handsome present, and for the loyalty which they had always shown towards him.

### A Book and its Story.

### THE VILLAGE AND THE DOCTOR.*

A STRANGE and sad interest attaches itself to the volume of short stories before us for review, inasmuch as its promising young writer lived only to correct the proofs, his death abruptly terminating what gave promise of being a brilliant literary career. No one reading the above-mentioned book can fail to see in it the undoubted elements of talent. James Gordon was a grandson of the late Dean Buckland, and the Buckland genius shows itself in his work. In his writings we notice a developed power of observation combined with a perfect lucidity of expression, a keen appreciation of humour, and an equal sense of pathos.

Through the short stories comprising the volume there is a vague connecting link—that is to say, the same characters are met with more than once; and, since characterisation is the writer's strong point, it only heightens the interest that the book must necessarily evoke. Among the stories "Medical Heretics" is perhaps the most attractive—not, perhaps, the most imaginative, but, to our mind, is the nearest to real life. It is the story of two old men, and it tells how one of them sought to restore his health otherwise than through the more regular medium of the medical consultant. It illustrates a particular phase in the life of certain uncultivated classes, and is humorous and entertaining.

"I be right down tired of this," said Neddie Thring. . . . Master Hook, who shared the cottage with Neddie, nodded his grey head from the chair on the opposite side of the fireplace. . . . Neddie continued—"That there doctor beant no good. 'E gives 'is best physic to the rich, and lets the club patients look after theirselves. . . . You mind, I went to the surgery this morning because I was so terrible queer; I thought my poor back would fall out of me. When the doctor sees me a-sitting there in 'is surgery, bent double and all tied in a knot with pain, 'e burst out a-laughing, and 'e says, says 'e, 'Look 'ere, Neddie,' says 'e, 'That club feast o' yesterday has done for you and settled on your chest . . .'" Hook looked depreciatingly towards Neddie, and said in a voice loud enough to be heard by Neddie's deaf ears—

"I ain't surprised nor sorry for yer, after all that you put away at the club feast. I wonders you are alive . . . What did the doctor give you?" enquired Hook. "Gimme? 'E gave me nothing—at least it was something in a blue bottle . . . when I come to smell it, I found there was no smell to it. And then I knew that Dr. Simons was taking me in. I puts down the bottle on the table, and I swears." "Swearing ain't no good," said Hook, although he often swore himself. . . . On the subject of sickness, acute and chronic, there was much sympathy between the two old men who lived together in the thatched cottage. The groans of the one inspired the pains of the other.

The conversation, as narrated above, was followed by a settled resolve on Neddie's part to improve on Dr. Simons' treatment on which he had showered the contempt of an unreasoning mind—and seek elsewhere to fill the club doctor's deficiencies. Rumours of the Great Cure-all Physician reached the old men's abode—of the celebrated Dr. Loftus, of gold chariot fame, who was effecting marvellous cures at Portsmouth: thither the two ultimately wandered, in gorgeous toilettes, as befitted the auspicious occasion. Dr. Loftus was a magician and inspired the mind of the populace. His "yellow chariot, drawn by four piebald horses, appealed to the artistic senses of the public, and the brass band, performing on the top of the vehicle, made noisy music for the ears of all. Twice daily Dr. Loftus paraded the town, and like the procession of Israelites walking round the town of Jericho blowing rams' horns." It did not take long for the town of Portsmouth to accept the great quack as their lost magician, guide, and friend.

Hook and Neddie arrived in Portsmouth, and pressed forward to the scene of action. "In the cabin of the state carriage Loftus fitted his leather garments and nodding plumes preparatory to commencing business. Through the hollow in the eye of Britannia, which ornamented the outside of the carriage, he contemplated with satisfaction the increasing number of the crowd." Later on, arrayed in his flowing Indian garments, the American physician held forth on the merits of his cure; brass drums and blatant instruments interspersing his remarks. After a popular, if not a strictly scientific explanation of the laws which govern health, the eloquent exponent continued: "I owe my life to this little phial. I have not long to live, but I should have been dead and buried long years ago but for this natural remedy. . . . As I saved myself, I'll save you. . . . What I can do for corruption, I can do for the teeth by painless American dentistry. I'm now going to draw teeth free and gratis by one of the greatest discoveries of the age—the painless dentistry of America." Dr. Loftus called attention to the fact that he had had offers from doctors and dentists for the secret of his discovery, which he had declined; one especial offer had embraced more sovereigns than there are days in the year.

"Ye fool; you ought to have closed with him," said a voice in the crowd. "That is where you make the mistake, my friend," said Loftus, answering the unknown voice with a shake of the head. "What would the poor do without Loftus? Who else would draw their teeth for nothing? Who else would cure their rheumatics and other complaints?" At the close of his able defence there was a rush for the platform where the philanthropical Doctor stood. "Some uncontrollable impulse stimulated the public to have their teeth extracted." . . . For the fun of the thing rich and poor submitted to the novelty. Hook, among others, painfully ascended the steps. With a practised eye the Doctor noted the old man's decrepitude. "I can see at a glance what is the matter with you, my man!" cried Loftus. . . . "You have the rheumatics in the back and legs. I'll put that matter right for you when all the teeth are drawn." . . . After the first operation Hook proudly descended the steps. "Not a morsel or bit did it hurt," he exclaimed. Then, later on, rid of his teeth, old Hook is cured in a miraculous way of his rheumatism. Loftus extends the frail old form on a bench and applies the restorative measures known only to himself.

The story here described we have reason to believe was taken from life; instances of temporary cures such as Hook's at the hands of charioted quacks have already come before the public, though not handled deftly in print as the present writer handles them.

Hook went up the steps to Dr. Loftus' side with crippled limbs; after the interview he danced a jig on the chariot boards, and descended the stairs minus his props (p. 277). "If the old man ever wants a stick again, I will give him a stick of gold," said the exultant magician.

And Loftus displayed to the public a tray on which dozens of brilliantly-labelled bottles rested side by side. "That is the remedy in neat bottles!"

"The balm of rheumatism," the mystic remedy, price 2s. 6d., was instantly purchased in fabulous quantities by the awe-struck crowd, and bottle after bottle was sold.

"Another poor sufferer cured," Loftus announced to the crowd later on. "James Hook, of Silford, is his name." The people took up the cry and shouted, "Cured! cured! cured!" Like the verdict of a jury, the cry echoed and re-echoed through the yard.

For the sequel to old Hook's cure, and for further details of Dr. Loftus' career, we refer our readers to the book itself and to its humorous and strangely realistic descriptions.

*By James Gordon. (London: Methuen and Co. 1897.)

### The Victoria Commemoration Club.

The accompanying charming illustrations introduce our readers, who have not yet personally visited it, to the Victoria Commemoration Club for Nurses and Associated Workers. The club occupies a delightfully open site, and is centrally and conveniently situated. The charming turret windows look down Southampton Street to the Strand, whilst others in the principal rooms face a large open space belonging to Covent Garden Market. Thus it will be seen that a bright and airy situation has been chosen which is almost unique in so busy a part of London. The club is entered by a handsome doorway, where a small porter receives the visitors, and has charge of the book, in which all visitors' names are inscribed. The club occupies the second, third, and fourth floors of the handsome premises known as The Hospital Building, 28 and 29, Southampton Street, Strand.

The visitor first enters the charming drawing-room shown in the illustration. No one can help being struck by the delightful scheme of colour, the airy brightness and comfort which meets the eye. The room is large, lofty, and has ample window space, the little turret window at the end enhancing the artistic and original effect. One can picture weary members seeking a comfortable corner amidst the cushions of the luxurious sofa, within reach of which stands the table covered with all manner of periodicals, professional and otherwise. Dainty little window blinds of tinted silk soften the light which enters the fine windows, and beautiful rugs bring the colours into harmony. This room, in common

The Drawing Room.

The Tea and Luncheon Room.

with the whole of the building, is lighted with electric light.

On the same floor as the drawing-room are the secretary's apartments. The office is a most inviting room, artistically furnished and arranged with the many beautiful treasures belonging to its occupant, and an interview in so charming a spot is robbed of disagreeable chilling formality and made pleasant by the kind courtesy of the secretary herself.

On the upper floor is situated the restaurant, or tea and luncheon room shown in the illustration. The little picture is so complete that description is hardly necessary. The same pretty colouring adorns this apartment, and the same taste has been exercised in selecting the pictures and all the details. The china and glass used is dainty, and we have heard expressions of admiration at the tempting manner in which refreshments are served, at a cost as low as an A. B. C. shop, where certainly the serving is not appetising. Leading out of the dining-room is the writing and silence room, also illustrated. Here every convenience for writing is supplied—nice writing-paper, blotting-pads, and good pens—so rare, but so necessary an accessory of the writing table. Pretty little electric lamps will be seen to adorn the table, and the same sense of bright, fresh completeness prevails here as elsewhere.

On the same floor is the dressing-room, wherein all will be found complete for the toilet. A goodly row of hanging-hooks and stands for the parcels of weary shoppers testify to the thought for, and comprehension of, a nurse's wants. On the top floor, situated in the quietest portion of the building, is the lecture-room shown in the illustration. The platform is most convenient, and

THE WRITING AND SILENCE ROOM.

THE LECTURE ROOM.

the room in every way most suitable for its purpose. On the same floor are kitchen and offices, and the kitchen is by no means the least attractive portion of the club. A visit to it is quite enough to assure the members that they will secure clean and wholesome fare. Lastly, a word should be said for the lavatories, which are as adequately equipped as the rest of the establishment.

The illustrations, we imagine, will be quite eloquent witnesses to the really charming establishment, which has been instituted solely for the benefit of nurses and those ladies whose work leads them amongst the sick and needy. It has often been said that such a haven was needed for this large class of busy workers, but the very heavy expense of establishing such a club has alarmed well-wishers hitherto. It has been said that nurses and workers in the charity world do not understand co-operation, and prefer to go on without the anchorage they really need, not having the initiative to enter and support one for themselves. But this the promoters of the Victoria Commemoration Club do not believe. They think that to country nurses who visit the metropolis the boon of such an establishment must above all be apparent. Some place where parcels can be received, a restful hour secured, means of tidying up, securing apartments, receiving friends, attending lectures and brushing up knowledge, and making appointments with facility by means of the telephone is exactly what the country nurse above all needs. Then for the private nurse in London, what an economy of expenditure. It renders it possible for a nurse to live a comfortable and civilised life, and yet have the burden of the rent of a bed-room only to pay for. She can always turn to the club when she is unoccupied, secure her meals if she will, and have a room at her disposal in which she may well be proud of receiving her friends, whilst having the advantage of being within call by means of the telephone or by telegram, using the cheap telegraphic address of the club. Seeing the immense advantages the club offers, we cannot but think that nurses will do well to join as soon as possible, as numbers are to be limited, and already nearly all who join bring recruits, so delighted are they with their own experience. The entrance fee will come into force after June, which those joining now avoid. We anticipate the excellent secretary will be very busy as June approaches, finding apartments for country members desirous of catching a glimpse of our Queen and the attendant festivities of the Commemoration.

### Appointments.

The Hospital for Women and Children, St. Michael's Hill, Bristol.—Miss Jessie Southwell has been appointed Matron to the Hospital for Women and Children, St. Michael's Hill, Bristol. She was trained and certificated at St. Bartholomew's Hospital, and has taken the L.O.S. certificate. For the last eighteen months Miss Southwell has filled the post of night sister to the Chelsea Hospital for Women; practically combining with this office the work of assistant matron. She leaves Chelsea Hospital for Women accompanied by the heartiest wishes of both matron, medical, and nursing staff for her success in her new sphere of work.

City of London Hospital for Diseases of the Chest, Victoria Park, E.—Miss Beatrice Jones has been appointed to succeed Miss Hetherington as Matron at this hospital. Miss Jones was trained at St. Bartholomew's Hospital, and has since held the position of assistant matron at the Infirmary, Birmingham.

Yeovil Hospital.—Miss S. Harvey has been appointed to the Matronship of the Yeovil Hospital. Miss Harvey was trained at Guy's Hospital, having subsequently held the position of matron at the Reynard Hospital, Gainsborough, and at the Easingwold Hospital, Yorkshire, and that of assistant matron at the Metropolitan Convalescent Home, Walton-on-Thames.

### Nurses and the Medical Profession.

Dr. Joseph Bell, of Edinburgh, well known in the nursing world as the author of "Notes on Surgery for Nurses," is writing some articles in the *Scottish Medical and Surgical Journal* on "The Relation of the Trained Nurse to the Profession and the Public." Dealing, in the March number, with her relation with the profession, he says: "The private nurse may either enter a private hospital, join a nursing corporation or institution from which she is sent to private cases, or, lastly, start on her own hook and trust to getting her work from doctors or patients whom she knows. With the first and third of these there is generally no friction in their relation to the profession. The nursing-home nurse is really much in the same comfortable state of shared responsibility as when in her training hospital. Instead of a share in a ward and its work, she now has one or two patients of her very own, but the matron is there at her elbow, and the doctors are accustomed to the ways of the house, wield a benevolent despotism, and friction is minimised. The nurse again who, from her own lodgings, is ready at the call of her own doctors or her own clientèle of patients is on assured ground. She is probably experienced, and certainly is sent for only by those who know her character and her ways. She in her turn wields a despotic sceptre, and with her doctors there are no difficulties. Far otherwise is it with the young nurse fresh from hospital who, having attached herself to a nursing institution, is the first on the list to go to a case. Her relations both to the patient and to the doctor in charge may be absolutely new to her. Her relations to the patient will be noticed hereafter. Those to the doctor are our present subject. He may be one of her own hospital staff, or at least on the staff of some hospital; then probably all will go smoothly. He will know her training, and where it is lacking in the wisdom that is needed in a private house; he will make allowances, and she will be proud to take a hint. Far otherwise is it in many a country district, where the doctor left hospital before the new school of nursing was invented, and probably neither knows nor likes the new-fangled ways. All nurse's fine ideas of antiseptic treatment—sterilised towels and iodoform gauze—are met by cheerful disdain. He may even prefer to take his temperature for himself, and he is apt to ignore the observations of the smart young probationer. Friction may ensue unless forbearance is shown on both sides, but the most highly trained hospital nurse, and even house-surgeon, can learn many a wrinkle from the old practitioner. The nurse must avoid any word or even hint that might suggest criticism of the old man's ways; she had better never quote the hospital or its staff, and loyally do her best to maintain the position and credit of the family attendant. . . . In the cottage a district nurse's help may make the difference between a success and a failure. In the houses of the sick one trained nurse is quite equal to a second surgical assistant. But she must keep her own place, and be very careful never to presume to take that of the family doctor. . . . Let the profession of medicine loyally trust their nurses, educate them as freely as possible, see that they are neither over-worked or under-paid, and on their side let the nurses be obedient and gentle, and above all let them keep to their own duties. When a nurse usurps or encroaches on the work of a doctor she makes a grave error. Probably one of the most difficult of all relations is that of the trained nurse to the lady doctor. The nurse is probably older than her medical sister, for a nurse rarely finishes her training till she is twenty-seven, a doctor may get her diploma at twenty-two. It is less easy for the nurse to obey one of her own than one of the opposite sex, and the lady doctor is very apt to stand upon her dignity. The safest solution of the difficulty is to fit the very young lady doctor with an old motherly experienced nurse, while the elderly lady doctor may be trusted to snub and dominate a young probationer.

### For Reading to the Sick.

"THE TRIVIAL ROUND."

**Verses.**

IF in our daily course our mind
Be set to hallow all we find,
New treasures still, of countless price,
God will provide for sacrifice.

We need not bid for cloistered cell,
Our neighbour and our work farewell;
Nor strive to wind ourselves too high
For sinful man beneath the sky.

The trivial round, the common task,
Would furnish all we need to ask;
Room to deny ourselves; a road
To bring us daily nearer God.

Seek we no more; content with these
Let present rapture, comfort, ease,
As heaven shall bid them, come and go,
The secret this of rest below.

Only, O Lord, in Thy dear love
Fit us for perfect rest above;
And help us, this and every day,
To live more nearly as we pray. —*Keble.*

Despise not thou small things,
The soul that longs for wings
To soar to some great height of sacrifice too oft
Forgets the daily round,
Where little cares abound,
And shakes off little duties while she looks aloft.

Every hour that fleets so slowly
Has its task to do or bear;
Luminous the crown, and holy,
When each gem is set with care.
Do not linger with regretting,
Or for passing hours despond;
Nor, the daily toil forgetting,
Look too eagerly beyond.
—*A. A. Proctor.*

**Reading.**

Never fancy you could be something if only you had a different lot and sphere assigned to you. The very things that you most deprecate, as fatal limitations or obstructions, are probably what you most want. What you call hindrances, obstacles, discouragements, are probably God's opportunities.

How can you live sweetly amid the vexatious things, the irritating things, the multitude of little worries and frets, which lie all along your way, and which you cannot evade. You cannot at present change your surroundings. Whatever kind of life you are to live must be lived amid precisely the experiences in which you are now moving. Here you must win your victories or suffer your defeats. No restlessness or discontent can change your lot. Others may have other circumstances surrounding them, but here are yours. You had better make up your mind to accept what you cannot alter. You can live a beautiful life in the midst of your present circumstances.—*J. R. Miller.*

It is not by seeking more fertile regions where toil is lighter—happier circumstances, free from difficult complications and troublesome people—but by bringing the high courage of a devout soul, clear in principle and aim, to bear upon what is given to us, that we brighten our inward light, lead something of a true life, and introduce the Kingdom of Heaven into the midst of our earthly day. If we cannot work out the Will of God where God has placed us, then why has He placed us there?—*J. H. Thorn.*

### Notes and Queries.

**The contents of the Editor's Letter-box have now reached such unwieldy proportions that it has become necessary to establish a hard and fast rule regarding Answers to Correspondents. In future, all questions requiring replies will continue to be answered in this column without any fee. If an answer is required by letter, a fee of half-a-crown must be enclosed with the note containing the enquiry. We are always pleased to help our numerous correspondents to the fullest extent, and we can trust them to sympathise in the overwhelming amount of writing which makes the new rules a necessity. Every communication must be accompanied by the writer's name and address, otherwise it will receive no attention.**

*"The Trained Nurse."*

(189) Please tell me where I can get the March number of "The Trained Nurse," and what is its price? Stamped envelope enclosed.—*Nurse S.*

"The Trained Nurse" is published in America. The Scientific Press, 28 and 29, Southampton Street, Strand, is the sole agent in England Read our rules at the head of this column respecting replies by post.

*Private Nursing.*

(190) Can you tell me of an institution where nurses can obtain private cases, paying a percentage of fees received, and reside at their own home? —*Maternity Nurse.*

Have you had general training as well as training in monthly nursing? If you state your qualifications more fully, we should be better able to advise you.

*Derbyshire Royal Infirmary.*

(191) Will you kindly tell me if the Derbyshire Royal Infirmary is under the Local Government Board or is a private hospital?—*Sister A.*

The Derbyshire Royal Infirmary is a general hospital, supported by voluntary contributions. If the word "infirmary" led you to think it a State institution, we may explain that many old-established hospitals have retained this title instead of the more modern word "hospital."

*Ward Furniture.*

(192) Please tell me of a firm who will supply ward furniture. I want a good locker on the pedestal principle, and a medicine cupboard.—*Sister-in-Charge.*

Messrs. Debenham and Freebody, Wigmore Street, Messrs. Maple, and Messrs. Shoolbred, both in Tottenham Court Road, supply ward furniture, or if you want the newest thing in aseptic furniture, iron and glass, write to Messrs. Down Brothers, St. Thomas's Street, S.E., who have made a special study of the subject. We should recommend you, before ordering either locker or medicine cupboard, to visit one or two hospitals and examine for yourself the practical advantages of the various articles. The fittings and appliances are specially good at the Temperance Hospital, Hampstead Road, N.W., or the Poplar Hospital for Accidents, Blackwall, E.

*A Difficulty.*

(193) Please advise me. I was engaged last year to nurse a lady this March, in consequence of which I refused other cases, and was ready at the end of February to fulfil my engagement. But the baby was born in February, so another nurse had to be sent for, and when I wrote to ask if I should go on March 1st, I was told my services were not required. I have written for my fee, but have had no reply. What can I legally claim?—*Monthly Nurse.*

We understand that if you have full proof of your engagement you can claim full fees, but it is usual to take half fees in such cases, and we should advise you to apply for this through a lawyer at once.

*Indian Nursing Service.*

(194) Can you supply me with the pass list of the Indian Service examination? I have seen the papers submitted to candidates, but not the result. Stamped envelope enclosed.—*K. A.*

Please explain more fully what you mean. Any inquiries respecting the Indian Nursing Service should be addressed to the India Office, Whitehall, S.W. We cannot answer queries by post except in accordance with the regulations at the head of this column.

*"The Pro."*

(195) Please tell me in which number of THE HOSPITAL I shall find some verses headed as above? I think they appeared in 1891 or 1892. Can I get the back number from THE HOSPITAL Office?—*Yorkshire.*

We have handed your letter to the manager, and asked him to send you the number containing the verses.

*Melbourne Hospital.*

(196) Where could I get a pattern of the cap worn by the Melbourne Hospital sisters, of which you gave a picture in THE HOSPITAL last autumn?

You had better write to Miss Farquharson, the Matron, and ask if she will let you have a pattern of the cap.

*Dispensing.*

(197) I am shortly going abroad to work in the foreign mission field, and before I go shall have to learn dispensing. Could you recommend books to help me before I begin the practical part of the work?—*Fuh-ning.*

Read the article on this subject this week, in which a list of books is given.

*Medicine.*

(198) Can you recommend me some small book containing information respecting the different medicines, their probable effects, when to be administered, &c.? Also any fairly simple book on anatomy? Is Miss Hampton's book on nursing as useful a book of reference as any other, or can you recommend me a better, for private nurses particularly?—*Medicine.*

(1) Dr. Percy Lewis's "Theory and Practice of Nursing"; (2) "Elementary Anatomy and Surgery for Nurses," by W. McAdam Eccles; (3) Miss Hampton's book is excellent, but perhaps for private nursing Miss Stoney's "Practical Points in Nursing" will suit you best. All these books may be had from the Scientific Press, 28 & 29, Southampton Street, Strand, W.C.

## Figures and Tables

**Figure f1:**
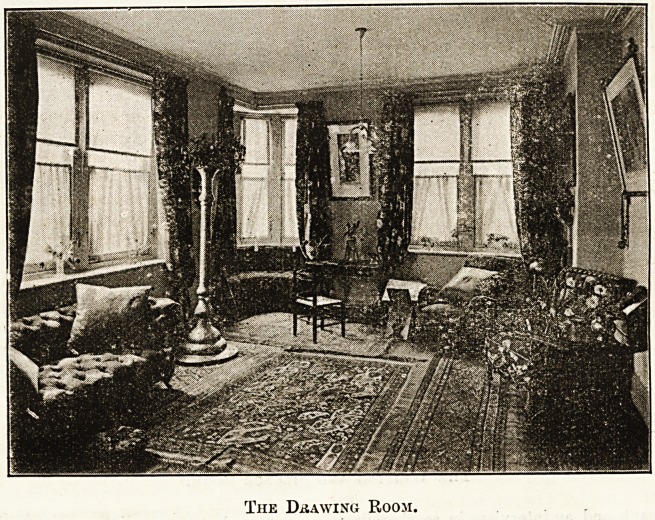


**Figure f2:**
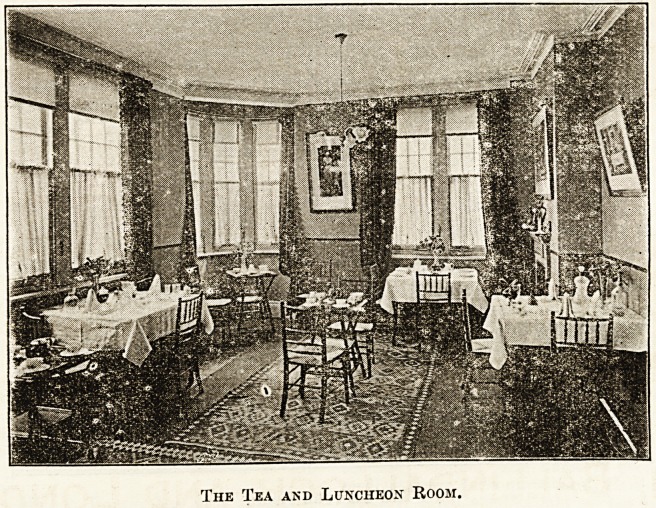


**Figure f3:**
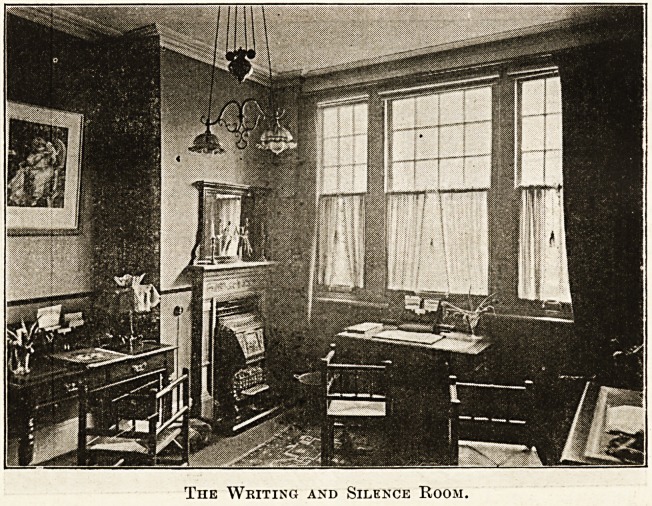


**Figure f4:**